# Comment on Mahajan, S.; Schmidt, M.H.H. Distinct Lineage of Slow-Cycling Cells Amidst the Prevailing Heterogeneity in Glioblastoma. *Cancers* 2023, *15*, 3843

**DOI:** 10.3390/cancers16020277

**Published:** 2024-01-09

**Authors:** Changlin Yang, Duane A. Mitchell, Loic P. Deleyrolle

**Affiliations:** 1 Adam Michael Rosen Neuro-Oncology Laboratories, Department of Neurosurgery, University of Florida, Gainesville, FL 32611, USA; 2 Preston A. Wells, Jr. Center for Brain Tumor Therapy, University of Florida, Gainesville, FL 32611, USA; 3 Department of Neuroscience, McKnight Brain Institute, University of Florida, Gainesville, FL 32611, USA

We greatly appreciate the interest, careful reading, and appraisal by Mahajan and Schmidt [1] of our research article titled “Slow-Cycling Cells in Glioblastoma: A Specific Population in the Cellular Mosaic of Cancer Stem Cells” [2].

This commentary provides an excellent overview of the challenges presented by glioblastoma (GBM), emphasizing the significance of cancer stem cells (CSCs) in tumor progression and recurrence. We are appreciative of the acknowledgment of the importance of our study in shedding light on the role of slow-cycling cells (SCCs) within the heterogeneous GBM cellular landscape.

GBM is indeed a formidable challenge, characterized by its aggressiveness, invasive nature, and resistance to conventional treatments. As you pointed out, the standard therapeutic approaches, including surgical resection, radiotherapy, and chemotherapy, have provided limited success, and patient prognosis remains extremely poor. The notion that a subset of cells, i.e., CSCs, plays a central role in tumor growth and recurrence is of great importance. The inherent heterogeneity within the CSC population, driven by numerous intrinsic and extrinsic factors, presents a formidable obstacle in understanding and treating this complex disease. The current editorial underscores the urgency of addressing the survival of CSCs following therapy, which has proven to be a significant factor contributing to the failure of various treatment regimens.

We appreciate your focus on slow-cycling cells (SCCs) within the context of CSCs and their role in the broader issue of tumor heterogeneity in gliomas. The discussion on SCCs is particularly timely and supported by multiple pieces of evidence from mouse models, xenograft assays, and single-cell transcriptomics, elucidating their involvement in glioma development and their propensity for resistance to treatment. As Mahajan and Schmidt accurately pointed out, the characterization of these cells is crucial, particularly understanding their unique characteristics and functions and how they differ from other glioma CSCs, all of which are integral to the quest for more effective therapies against GBM.

The recognition of SCCs through their elevated lipid metabolism and involvement in autophagy is a critical finding. These pathways have been recognized as mechanisms engaged by tumor cells to resist therapy in various cancer types. Mahajan and Schmidt’s emphasis on this aspect underscores the clinical implications of our study. The potential to target these pathways offers a promising approach to disrupt GBM. Strategies aimed at modulating lipid metabolism and autophagy in GBM cells could hold the key to enhancing the effectiveness of treatment regimens. This commentary not only highlights the significance of these findings but also emphasizes their therapeutic potential.

Our single-cell RNA sequencing analysis, leveraging this lipid metabolism signature, confirmed significant transcriptomic differences between SCCs and their counterparts, further substantiating the notion that SCCs represent a unique and distinct population or lineage within the glioma mass. Your comment on the limited cellular overlap between SCCs and other cell populations reiterates the significance of characterizing SCCs as a specific entity or cellular state. This absence of overlap not only deepens our understanding of the complex landscape of GBM but also suggests that SCCs may have distinct roles and functions that set them apart from the broader pool of classical CSCs. The incorporation of single-cell RNA sequencing in this context provided significant advancement in the quest to decipher the complexity of this disease, and we appreciate your attention to its methodological significance. This approach has the potential to uncover new aspects of SCC biology and their contribution to glioma development and progression.

Furthermore, your analysis of our study findings regarding the examination of the impact of marker expression on patient survival adds a crucial dimension to our comprehension of GBM complexity. The functional analysis of these different CSC populations related to disease state provides valuable insights into how they contribute to patient prognosis. As you pointed out, it is interesting to note that a high SCC score is associated with a poor outcome. These findings emphasize the clinical relevance of understanding SCCs, as they can potentially inform patient stratification, treatment strategies, and clinical management.

In conclusion, your in-depth analysis of our work underscores the critical findings we have presented. We concur with your observation that SCCs represent a unique population within GBM and display distinctive characteristics, particularly regarding molecular markers, cell lineage, infiltrative ability, metabolic properties, and resistance to treatment. The identification of SCCs as a crucial component of GBM heterogeneity and disease presentation opens the door to exploring novel therapeutic strategies, including targeting specific signaling pathways like lipid metabolism and autophagy.

We are encouraged by your perspective on our research and your suggestion of combinatorial therapies to address this challenging disease. Your editorial serves as a valuable contribution to the ongoing discussion surrounding GBM, CSCs, and SCCs, and it will undoubtedly guide future investigations and clinical interventions in the field.

Once again, we would like to extend our sincere appreciation for your thorough review of our work and your thoughtful commentary. Your support and insights are invaluable to us, and we enthusiastically anticipate further discussions and collaborations within the scientific community.
